# The EuroFlow PID Orientation Tube for Flow Cytometric Diagnostic Screening of Primary Immunodeficiencies of the Lymphoid System

**DOI:** 10.3389/fimmu.2019.00246

**Published:** 2019-03-04

**Authors:** Mirjam van der Burg, Tomas Kalina, Martin Perez-Andres, Marcela Vlkova, Eduardo Lopez-Granados, Elena Blanco, Carolien Bonroy, Ana E. Sousa, Anne-Kathrin Kienzler, Marjolein Wentink, Ester Mejstríková, Vendula Šinkorova, Jan Stuchly, Menno C. van Zelm, Alberto Orfao, Jacques J. M. van Dongen

**Affiliations:** ^1^Department of Immunology, Erasmus MC, Rotterdam, Netherlands; ^2^Department of Pediatrics, Laboratory for Immunology, Leiden University Medical Center, Leiden, Netherlands; ^3^Department of Paediatric Haematology and Oncology, Second Faculty of Medicine, Charles University and University Hospital Motol, Prague, Czechia; ^4^Department of Medicine, Cancer Research Centre (IBMCC, USAL-CSIC), Cytometry Service (NUCLEUS), University of Salamanca (USAL), Institute of Biomedical Research of Salamanca (IBSAL), Salamanca, Spain; ^5^Biomedical Research Networking Centre Consortium of Oncology (CIBERONC), Instituto de Salud Carlos III, Madrid, Spain; ^6^Institute of Clinical Immunology and Allergology, St Anne‘s University Hospital, Brno, Czechia; ^7^Immunology, Universitario La Paz, Madrid, Spain; ^8^Laboratory for Clinical Biology and Hematology, University Hospital Ghent, Ghent, Belgium; ^9^Faculdade de Medicina, Instituto de Medicina Molecular, Universidade de Lisboa, Lisbon, Portugal; ^10^BRC-Translational Immunology Lab, University of Oxford, Oxford, United Kingdom; ^11^Department of Immunology and Pathology, Central Clinical School, Monash University, Melbourne, VIC, Australia; ^12^Department of Immunohematology and Blood Transfusion, Leiden University Medical Center, Leiden, Netherlands

**Keywords:** flow cytometric immunophenotyping, primary immunodeficiencies, automated gating strategy, standardization, EuroFlow

## Abstract

In the rapidly evolving field of primary immunodeficiencies (PID), the EuroFlow consortium decided to develop a PID orientation and screening tube that facilitates fast, standardized, and validated immunophenotypic diagnosis of lymphoid PID, and allows full exchange of data between centers. Our aim was to develop a tool that would be universal for all lymphoid PIDs and offer high sensitivity to identify a lymphoid PID (without a need for specificity to diagnose particular PID) and to guide and prioritize further diagnostic modalities and clinical management. The tube composition has been defined in a stepwise manner through several cycles of design-testing-evaluation-redesign in a multicenter setting. Equally important appeared to be the standardized pre-analytical procedures (sample preparation and instrument setup), analytical procedures (immunostaining and data acquisition), the software analysis (a multidimensional view based on a reference database in Infinicyt software), and data interpretation. This standardized EuroFlow concept has been tested on 250 healthy controls and 99 PID patients with defined genetic defects. In addition, an application of new EuroFlow software tools with multidimensional pattern recognition was designed with inclusion of maturation pathways in multidimensional patterns (APS plots). The major advantage of the EuroFlow approach is that data can be fully exchanged between different laboratories in any country of the world, which is especially of interest for the PID field, with generally low numbers of cases per center.

## Introduction

Primary immunodeficiencies (PID) of the lymphoid system are rare inherited disorders with heterogeneous clinical presentations (1, 2). Most patients have clinical manifestations of immune dysfunction such as recurrent infections (early in life), and autoimmunity frequently causing irreversible organ damage in case of delayed diagnosis. As a consequence fast and efficient diagnostic screening is required. Advanced multicolor flow cytometry serves on this need. Flow cytometric immunophenotyping of T, B, and NK cells is the classically recommended method in the diagnostic work-up in case of a suspicion of PID of the lymphoid system. The complete diagnosis and classification consists of stepwise screening and subsequent characterization for numerical alterations in lymphocyte (sub) populations, detection of functional defects, and functional assays. However, lack of standardization and the rarity of PID has so far complicated a common strategy in PID diagnostics.

The introduction and the availability of next generation sequencing (NGS) based on targeted panel sequencing or whole exome sequencing (WES) with a filter for PID genes has an important impact on PID diagnostics in identification of the variants in known PID genes (3, 4). Moreover, it contributes to the broadening of the clinical spectrum of known PIDs. Finally, WES and whole genome sequencing (WGS) allows the identification of genetic defects in new PID candidate genes. However, the turnaround time is relatively long (i.e., a couple of months in a routine diagnostic setting) in contrast to flow cytometry, which already provides complete insight into the composition of the lymphoid compartment within a day. For correct interpretation of NGS data, it is crucial that the immunophenotype is known. Furthermore, flow cytometry can play an important role in the functional validation of genetic variants to evaluate the impact on the immune system. Altogether, this illustrates that both NGS and flow cytometry are valuable tools in PID diagnostics.

In this study, we developed a PID screening and orientation tube which allows fast and robust detection and enumeration of the lymphocyte subsets. It is important to notice that more than 70% of all PID concern inborn defects in the lymphoid system. Orientation in an early phase of the diagnostic process forms the basis for consecutive diagnostics, treatment, and clinical management. Therefore, we need a PID screening and orientation tube (PIDOT) which allows dissection of especially the lymphoid compartment in peripheral blood with full standardization to allow international comparability of results.

## Methods

### Patient and Control Samples

Peripheral blood samples were collected from 250 healthy controls divided into 14 age groups: cord blood (*n* = 15), neonatal blood (*n* = 16), 1–5 month (*n* = 12), 5–11 m (*n* = 7), 12–24 m (*n* = 30), 2–4 years (*n* = 35), 5–9y (*n* = 28), 10–17y (*n* = 18), 18–29y (*n* = 31), 30–39y (*n* = 15), 40–49y (*n* = 12), 50–59y (*n* = 10), 60–69y (*n* = 10), >70y (*n* = 11). Healthy controls were selected as having no signs or suspicion of immunological or hematological diseases (including an abnormal infection rate or a known history of allergies). All individuals were vaccinated following similar national vaccination schedules (European Center for Disease Prevention and Control; *http://vaccine-schedule.ecdc.europa.eu/Pages/Scheduler.aspx)*. They were enrolled at the different EuroFlow laboratories after informed consent was provided by each subject, their legal representatives, or both, according to the Declaration of Helsinki. In addition 99 patients with a genetically defined PID were collected according to the local medical ethics regulations of the participating centers. All samples were collected after informed consent was provided by the subjects, their legal representatives, or both, according to the Declaration of Helsinki. The study was approved by the local ethics committees of the participating centers [University of Salamanca, Salamanca, Spain (USAL-CSIC 20-02-2013); Charles University, Prague, Czech Republic (15-28541A); Erasmus MC, Rotterdam, The Netherlands (MEC-2013-026); University Hospital Ghent, Belgium (B670201523515) and St Anne‘s University, Brno, Czech Republic(METC 1G2015)].

### Assessment of Absolute Numbers of B, T, and NK Cells

The absolute number of lymphocytes (B, T, and NK cells) was determined either in a separate TrueCount (BD) tube with anti-CD45 PerCP alone or BD Multitest™ CD3/CD16+CD56/CD45/CD19 or it was determined by hematological analyser as a part of a diagnostic workup.

### Design of the PID Screening Tube

The PID screening tube was designed to assess the composition of the lymphoid compartment in a single 8-color labeling for guiding diagnosis of PID patients via detecting all relevant subpopulations. To this end, at certain fluorochrome positions two markers, i.e., a B-cell and a T-cell marker were combined of which is absolutely secured that they are exclusively expressed on only one of the subsets. So, two similarly labeled antibodies defining two distinct populations are mixed. CD19 was combined with TCRγδ, CD4 with IgM, and CD8 with IgD. CD16 and CD56 were already combined on the same channel for detection of NK cells. In addition to the above mentioned markers, we included CD3, CD45, CD27, and CD45RA. The optimal combination of clones and fluorochromes was reached after four rounds of testing in the participating centers (Supplementary Table 1). In order to achieve higher sensitivity to low abundant cell types, we used a lyse-stain-wash-fix protocol and the antibody staining time was increased to 30 min (6). This optimized staining procedure yielded more acquired cells (lymphocyte event counts were in average 2.89 × 10^e5^ in healthy controls and 1.32 × 10^e5^ in PID patients) per sample with less non-leukocyte particles (less debris), and yielded higher median fluorescence intensity (MFI) patterns for several antibodies (Figure S1). The data were acquired on BD LSRII or BD FACSCanto II instruments with the standard EuroFlow instrument settings (7). For data analysis, the Infinicyt software (Cytognos SL, Salamanca, Spain) was used in parallel to local data analysis software programs FACS Diva (BD) and FlowJo (FlowJo, LLC, Ashland, OR, USA). Infinicyt software is a commercially available product from Cytognos SL (Salamanca Spain) and a free-dowload demo version is available at www.infinicyt.com.

### Analysis of Lymphocyte Subsets in Healthy Controls

The lymphoid PID screening tube was used for analysis of 250 healthy controls of different ages to define the patterns and to set the reference for the database. All samples were analyzed in conventional analysis software programs (FlowJo and FACSDiva) and using Infinicyt Software. In both software packages the same analysis strategy was followed for definition of the lymphocyte subsets. Percentile ranges were calculated for each age group, for unified overview in Figure S2 values in patients compared to two or five standard deviations of controls were used.

### Analysis of Genetically Defined PID Patients

Next we tested the lymphoid PID screening tube on 99 genetically defined PID patients. The patients were classified according to the IUIS classification, which divides PID into 8 categories. First, the absolute number and relative frequencies of the lymphocyte subset populations were determined in all patients. This data set formed the basis for development of our new approach for flow cytometry in PID.

### Statistical Analyses

Mean and range values were calculated for all continuous variables using the SPSS statistical software (SPSS software v23, IBM, Armonk, NY). Data files from 50 healthy donors were merged and lymphocytes subsets of interest identified using bivariate plots (Figure). This analysis was used to define in an n-dimensional space the best principal component analysis 1 (PC1) vs. PC2 representation to discriminate these subsets using the Infinicyt software. The PCA representation of the data is graphically summarized in 2 × Standard Deviation (SD) curves to be used as a reference for supervised automatic analysis of the samples (APS view; **Figures 3**–**5**). In order to graphically display an overview of abnormalities found in a PID group adjusted to age, we calculated a relative distance from age matched healthy controls for each value in each PID case as a number of standard deviations (SD) from healthy controls. Values below−2 SD or above 2 SD are considered abnormal. Repeatedly abnormal values (in a given PID disease group) are plotted in supplementary figures. Calculations and graphic displays of the discriminating parameters (Supplementary Figure 2) were created using R-project/ Bioconductor *http://www.r-project.org* and Microsoft Excel for Mac 2011 (Redmont, WA, USA).

## Results

### Multidimensional Analysis of the EuroFlow PID Orientation Tube

**Figure 1:**
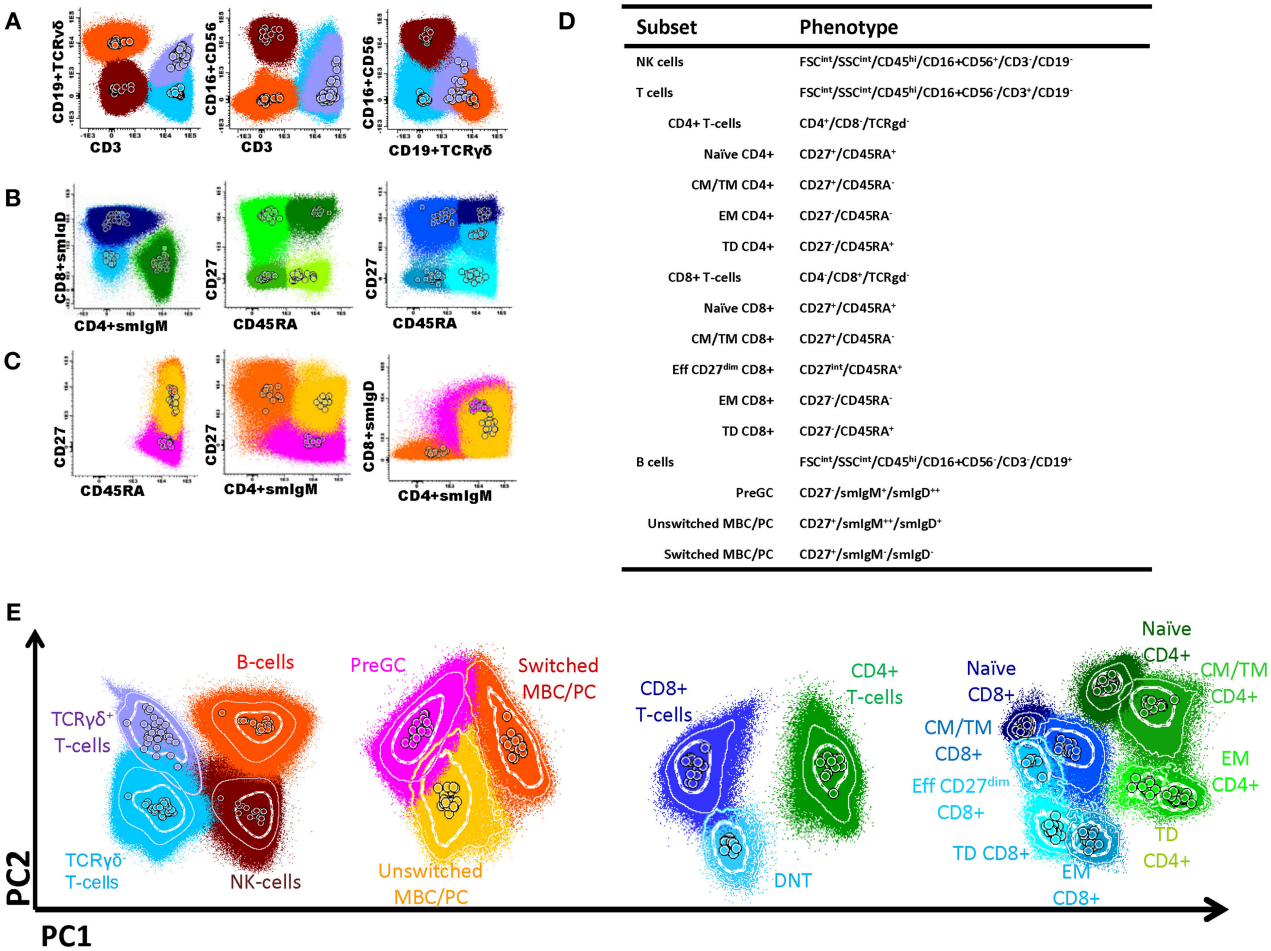
Flow cytometric analysis of B- and T-cell populations using the EuroFlow PID screening tube, 50 healthy controls are shown in the same plot, median of each gated subset is shown as a circle. **(A)** After gating leukocytes as CD45+ and lymphocytes on FSc and SSc, the markers CD3, CD19 in combination with TCRγδ and CD16+56 were used to define B-cells (orange); TCRγδ+ T-cells (lilac); TCRγδ- T-cells (blue); and NK cells (brown). **(B)** The T-cell subsets were further subdivided into naïve (CD27+CD45RA+; dark green), central memory/transitional memory (CM/TM; CD27+CD45RA-; bright green), effector memory (EM; CD27-CD45RA-; green) and terminally differentiated (TD; CD27-CD45RA+; light green) CD4+ T cells and into naïve (CD27+CD45RA+; purple), CM/TM (CD27+CD45RA-; dark blue), EM (CD27-CD45-; pale blue), and TD (CD27-CD45RA+; turqoise) CD8+ T cells. Also, as previously reported (5), some effector CD8+ T-cells showed dim CD27 positivity (EffCD27dim; CD27int-CD45RA+; blue). CD4/CD8 double negative T-cells are indicated in light blue. **(C)** B-cell subsets could be further subdivided into pre germinal center (PreGC; IgM^+^IgD^+^CD27^−^; orange) unswitched memory B-cells/plasma cells (Unswitched MBC/PC; IgM^+^IgD^+/−^CD27^+^; yellow), switched memory MBC/PC (IgM^−^IgD^−^CD27^+^; pink). **(D)** Definition and hierarchy of the defined subsets. **(E)** Multidimensional view (APS view) based on the most discriminating parameters for lymphocytes, B-cell, T-cells, and T-cell subsets.

In this study, we aimed to advance flow cytometric immunophenotyping of PID patients by linking the flow cytometric data to potential immunological defects and by incorporating this approach into the diagnostic process. To this end, we designed a PID orientation tube (PIDOT) (8 colors; 14 parameters) that allowed the analysis of all main lymphocyte subpopulations in a single standardized and validated tube (Table 1, Figure 1). After gating leukocytes as CD45+ and lymphocytes on FSc and SSc, the markers CD3, CD19 in combination with TCRγδ and CD16+56 were used to define B-cells, TCRγδ+ or TCRγδ- T-cells and NK cells (Figure 1A). The T-cell subsets were further subdivided into naïve, central memory (CM)/transitional memory (TM), effector memory and terminally differentiated (TD) CD4+/CD8+ T cells (Figures 1B,D); for CD8+ T-cells one extra population, effector CD27^dim^ was defined. Also CD4-CD8- (double negative) T-cells were defined (5). B-cell subsets were further subdivided into pre germinal center B-cells (PreGC), unswitched memory B-cells (MBC) or plasma cells (PC), and switched MBC Figures 1C,D) (8, 9). The total set and hierarchy of lymphocyte subsets that was identified is listed in Figure 1D.

**Table 1:** Composition of the EuroFlow PID Screening tube and information of monoclonal antibodies used in the PID screening tube including volumes, clones, and suppliers.

**BV421**	**BV510**	**FITC**	**PE**	**PerCP-Cy5,5**	**PC7/PE-Cy7**	**APC**	**APC-H7**
CD27	CD45RA	CD8	CD16	CD4	CD19	CD3	CD45
		IgD	CD56	IgM	TCRgd		
**Marker**	**Fluorochrome**	**Clone**	**Source**	**Catalog number**	**μl/test**		
CD3	APC	SK7	BD Biosciences	345767	2.5		
CD4	PerCPC5.5	SK3	BD Biosciences	332772	7		
CD8	FITC	SKI	BD Biosciences	345772	5		
CD16	PE	3G8	BD Biosciences	555407	5		
CD19	PECy7	J3-119	Beckman Coulter	IM3628	5		
CD27	BV421	M-T271	BD Biosciences	562513	1		
CD27 alternative	BV421	O323	BioLegend	302823	1		
CD45	APCH7	2D1	BD Biosciences	641417	2		
CD45RA	BV510	HI100	BD Biosciences	563031	2.5		
CD45RA alternative	BV510	HI100	Biolegend	304141	2.5		
CD56	PE	C5.9	Cytognos	CYT-56PE	5		
SmIgD	FITC	IA6-2	BioLegend	348205	1.25		
SmIgM	PerCPCy5.5	MHM-88	BioLegend	314511	2		
TCRγδ	PECy7	11F2	BD Biosciences	649806	1		

To offer intuitive and fast interpretation of the complete lymphoid compartment we developed a new analysis and visualization strategy for the PIDOT using a principle component analysis based multidimensional view (APS graph). First, reference plots were generated using a set of 30 samples of healthy donors in Infinicyt software. The lymphocyte populations were manually analyzed and subsequently, the most discriminating projection into a single APS graph was determined (Figure 1E).

### Reference Values and Database

**Figure 2:**
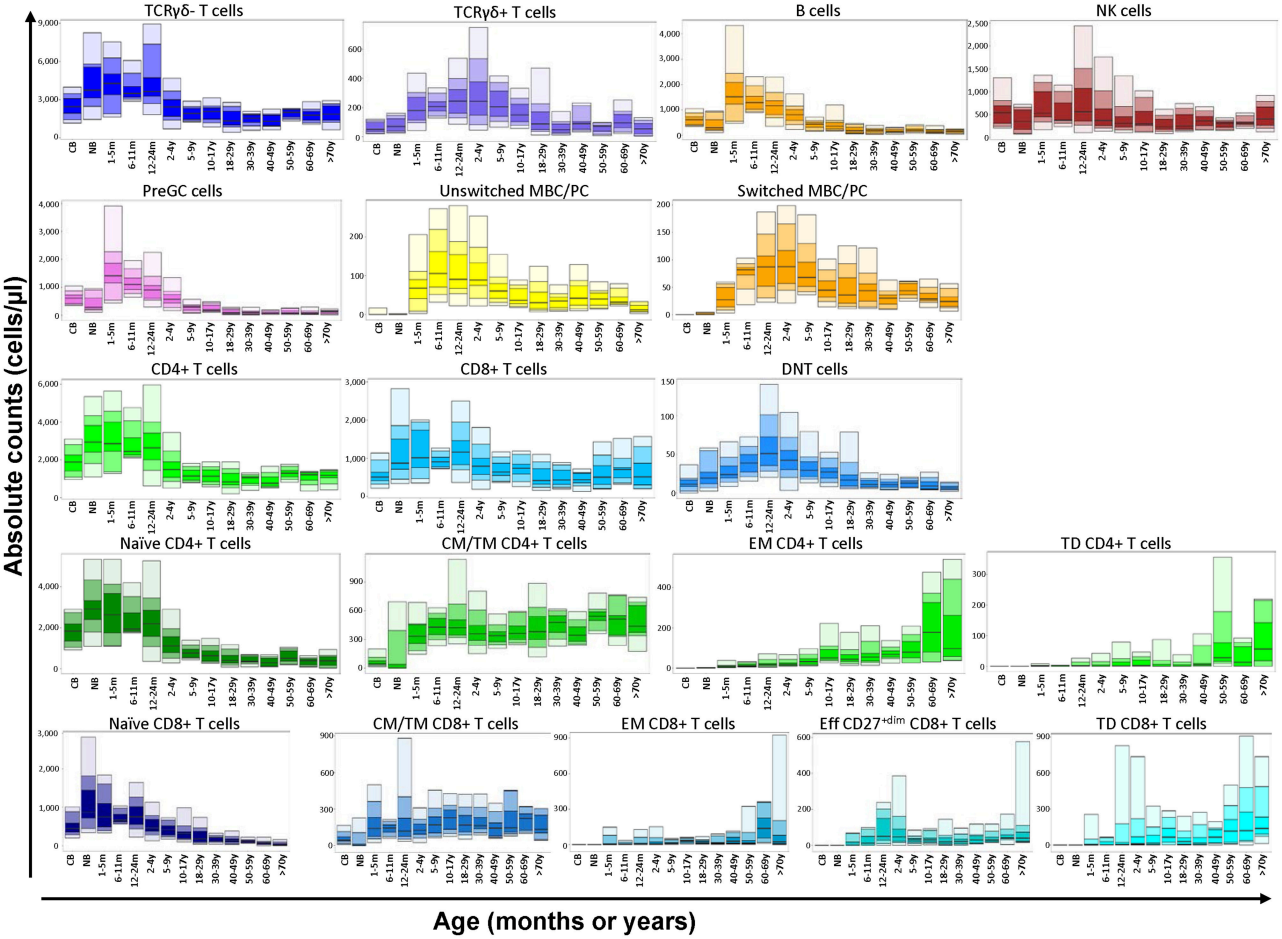
Flow cytometric analysis of B- and T-cell populations using the EuroFlow PIDOT in 250 healthy controls in 14 different age ranges. All values of this reference data set are displayed as bar graphs representing the median, minimum, maximum, and p10, p25, p75, and p90 percentiles. For data visualization package gplot2 for the statistical language R was used (10).

Subsequently, this analysis strategy and visualization was tested on 250 healthy controls in 14 different age ranges, which resulted in a unique reference data set of all lymphocyte subsets. All values of this reference data set are displayed as bar graphs representing the median, minimum, maximum, and p10, p25, p75, and p90 percentiles in Figure 2. Finally, 99 genetically defined PID patients were analyzed to study the performance of the EuroFlow PIDOT (Table 2).

**Table 2:** Frequency of patients with inborn errors of immunity showing defects of the major subsets identified in the EF PIDOT, as compared to age-reference values (Summary of Supplementary Tables 2, 3).

	**Any T cell subset**	**Any B cells subset**	**NK cells**	**Any of them**
SCID (*n* = 24)	100%	100%	25%	100%
IL2Rg	6/6	6/6	5/6	6/6
IL7R	1/1	1/1	0/1	1/1
RAG1	8/8	8/8	1/8	8/8
RAG2	5/5	5/5	0/5	5/5
DCLRE1C	3/3	3/3	0/3	3/3
NHEJI	1/1	1/1	0/1	1/1
CID (*n* = 12)	58%	83%	25%	100%
CD40L	1/6	6/6	0/6	6/6
ZAP70	3/3	1/3	0/3	3/3
DOCK8	2/2	2/2	2/2	2/2
BCL10	1/1	1/1	1/1	1/1
CID with syndromic features (*n* = 20)	70%	60%	10%	75%
WASp	3/3	3/3	0/3	3/3
ATM	5/6	4/6	0/6	5/6
Di George	3/6	1/6	0/6	3/6
STAT3	1/2	2/2	1/2	2/2
NEMO	1/2	1/2	0/2	1/2
PNP	1/1	1/1	1/1	1/1
PAD (*n* = 16)	31%	100%	25%	100%
BTK	1/10	10/10	1/10	10/10
PIK3CD	4/5	5/5	3/5	5/5
AID	0/1	1/1	0/1	1/1
Disease of immune dysregulation (*n* = 10)	70%	60%	10%	90%
Syntaxin	1/1	1/1	0/1	1/1
FAS	5/5	2/5	1/5	5/5
XLP	0/1	1/1	0/1	1/1
CD27	1/1	1/1	0/1	1/1
CTPS1	0/2	1/2	0/2	1/2
Defects of phagocytes or function (*n* = 10)	30%	60%	40%	70%
CGD	1/5	3/5	1/5	3/5
GATA2	2/5	3/5	3/5	4/5
Defects innate immunity (*n* = 3)	67%	67%	33%	67%
STAT1	1/1	1/1	1/1	1/1
WHIM	1/1	1/1	0/1	1/1
IRAK4	0/1	0/1	0/1	0/1
Complement deficiencies (*n* = 4)	0%	0%	0%	0%

*Results expressed as percentage of patients showing absolute counts below the lower limit of normality, compared to age-reference values obtained from 250 healthy donors analyzed with the same protocol. SCID, Severe Combined Immunodeficiency; CID, Combined Immunodeficiency; PAD, Predominantly Antibody Deficiency*.

### PID With Absence or Strong Reduction in One or More Lymphocyte Subsets

The two main categories of PID with absence or strong reduction in one or more lymphocyte subsets are SCID and agammaglobulinemia with absent T (NK) and/or B-cells.

#### SCID

**Figure 3:**
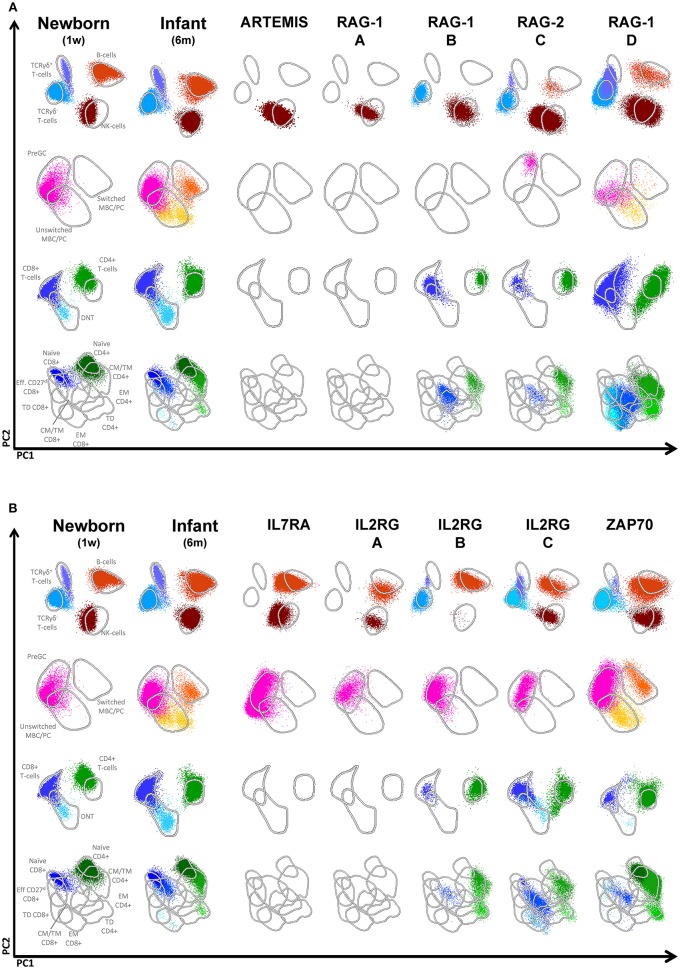
Flowcytometric analysis of B- and T-cell populations using the EuroFlow PID screening tube on controls and patients with SCID. From the top down, APS plots of gated lymphocytes, B-cells, CD3+ T cells and TCRgd- T cells are shown. Lines depict a 2 standard deviation boundary of all controls combined. **(A)** Multidimensional views of all lymphocyte subsets of a newborn, an infant, one Artemis-deficient and four RAG-deficient SCID patients. **(B)** Multidimensional views of all lymphocyte subsets of a newborn, one IL7RA-deficient, three IL2RG-deficient and a ZAP70-deficient patient.

Absence or strong reduction of lymphocyte subsets (B, NK, CD4, and CD8 T-cells) can be easily established by comparison of absolute numbers of lymphocyte subsets of patient vs. age-matched reference values. We analyzed patients with RAG1 (*n* = 8), RAG2 (*n* = 5), Artemis (*n* = 3), IL2RG (*n* = 6), IL7RA (*n* = 1), and ZAP70 (*n* = 3) deficiencies. In all patients the CD3-positive T-cells were strongly reduced, except for one patient with a RAG2 deficiency who presented with a high number of T-cells. In ZAP70 deficient patients, CD8-positive T-cells were reduced and in one of them also the CD4-positive T-cells, although to a lesser extent. NK cells, however, show a more heterogenous/variable pattern. This illustrates that NK cell numbers cannot straightforwardly be used for classification and it supports the idea to leave out NK cells for classification (11, 12). In addition to evaluation of the absolute counts, the APS views provide insight into the distribution of the lymphocyte subsets. RAG deficiencies can give a broad spectrum of clinical and immunological phenotypes. This partly depends on the type of mutation and the residual V(D)J recombinase activity (13–15). On top of that, the same mutation can also be associated with clinical heterogeneity (14). This phenomenon is also reflected in the APS profiles of RAG deficiencies (Figure 3A). RAG1, RAG2 as well as Artemis deficiencies with a null mutation will result in complete absence of B and T cells (RAG-1 patient A in Figure 3A). However, in case of a leaky or hypomorphic mutation T–cells are present (RAG-1 patient B in Figure 3A), which are in this case all TCRγδ- and had a memory or effector T-cell phenotype. In such situation the origin of T-cells need to be determined to investigate whether these T-cells are autologous or from maternal origin. A third pattern that can be seen is the presence of both T and B cells (RAG patient C and D). In both cases both TCRγδ- and TCRγδ+ T-cells are present (with a memory or effector phenotype) and the B-cells were mainly naïve or natural effector. No switched memory B cells or plasma cells were detected. So, the hallmark for RAG deficiencies with residual T-cells, which is characteristic for Omenn Syndrome, is absence or strong reduction of naïve CD4 and CD8 T-cells.

In IL2RG, IL7RA and ZAP70 deficiencies B-cell numbers were normal. IL2RG and IL7RA deficiency have in common that all B-cells have a naïve phenotype, which is in line with the fact that T-cell help is lacking for further differentiation (Figure 3B). In the ZAP70 deficiency some natural effector and switched memory B-cells are present. In case T-cells were present in patients with IL2RG deficiency, they had a memory phenotype (IL2RG B and C, Figure 3B).

### Agammaglobulinemia

**Figure 4:**
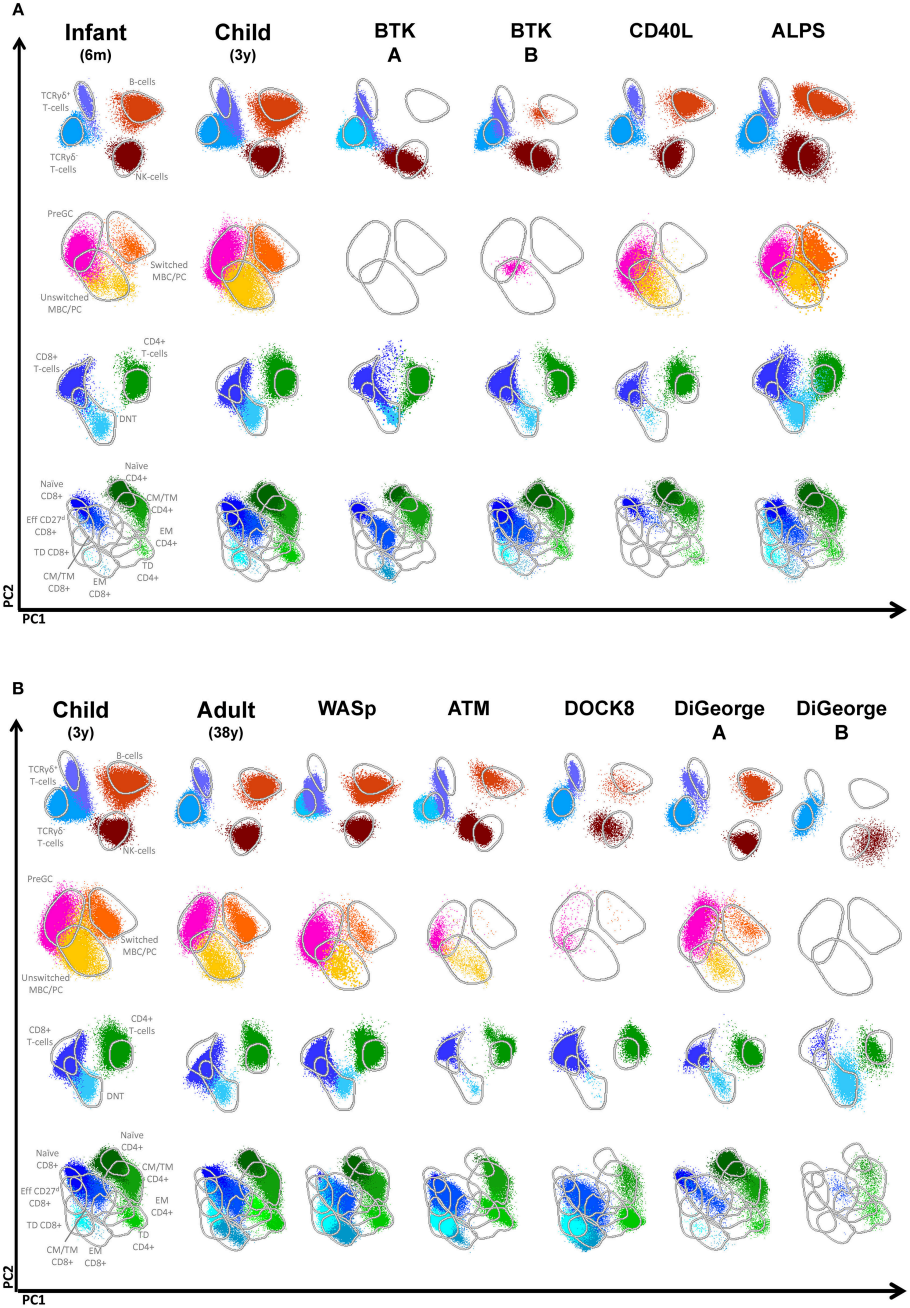
Multidimensional views of all lymphocyte subsets in healthy controls and PID patients. **(A)** Multidimensional views of all lymphocyte subsets of two BTK-deficient patients, a CD40L deficiency, a patient with ALPS due to a mutation in *FAS* and healthy infants of 6 months and 3 years. **(B)** Multidimensional views of all lymphocyte subsets of single examples of patients with Wiskott Aldrich syndrome (WASp), Ataxia Telangiectasia (ATM), DOCK8 deficiency and two patients with DiGeorge syndrome.

A second clear cut example in which lymphocyte subset analysis is highly informative in PID diagnostics is absence of B-cells in the 10 patients with X-linked agammaglobulinemia. The absolute number of B-cells is strongly reduced or the B-cells are even absent. As shown by the APS plots, if B-cells are present, they only have a naive phenotype (Figure 4A). The advantage of this approach is that on top of the maturation pathway that can be visualized with the APS plots, novel information can be obtained. The expression level within the naïve B-cells is shifted, indicating that the phenotype of this population also differs from normal.

### Disturbed Distribution Patterns of Lymphocyte Subsets in PID

In certain PID a specific distribution of lymphocyte subsets can be observed, which serves as a hallmark of the disease. A genetic defect in CD40L results in disturbed B-T interaction and consequently in reduced generation of (switched) memory B-cells that are T-cell dependent (16). As shown in a representative case, patients with CD40L deficiency have normal numbers of total B-cells, but they mainly consist of naïve or unswitched memory B-cells (Figure 4A). A second example is autoimmune lymphoproliferative disease (ALPS) mainly caused by mutations in FAS and FASL, which is characterized by the presence of a high frequency of TCRαβ+CD4-CD8- T-cells (i.e., double negative T-cells) (Figure 4A, Figure S2). Disturbed distributions of lymphocyte subsets or combinations of lymphocyte subsets can also be observed in other PID cases such as patients with WAS, ATM, DOCK8 deficiency, or DiGeorge syndrome (Figure 4B). However, these altered distribution profiles must be interpreted in the context of age-matched healthy controls. Therefore, correct interpretation relies on both patterns and absolute numbers.

We integrated the data of the total cohort of genetically-defined PID patients and the total set of healthy controls to define which lymphocyte subsets were abnormal (i.e., the absolute counts below the lower limit of normal, compared to age-reference values) and calculated the percentage of patients showing abnormal values (Table 2 and Supplementary Tables 2, 3). Per disease category the frequency of patients with defects in any of the lymphoid subsets detected with the EuroFlow PIDOT was determined. Naïve CD4, naïve CD8, and swiched memory B-cells were the most frequently aberrant populations (Figure S2). For example, in the category SCID all patients had aberrancies in any of the T-cell subpopulations or in any of the B-cell populations. More specifically, in all patients naïve T cells as well as unswitched and switched memory B-cells were reduced. Supplementary Tables 2, 3 give a further break down per T and B-cell subset. In other PID categories similar characteristic abnormalities were observed. For CID and PAD it was expected that aberrancies in lymphoid subsets are expected, but this dataset shows that also in patients with immune dysregulation and defects in phagocyte and innate immunity aberrancies were found with high frequencies. This illustrates that the PIDOT is a powerful tool to detect aberrancies in a broad range of PID with lymphocyte defects. As expected the PIDOT did not give any abnormalities in complement deficiencies, since these PIDs do not display any lymphocytes' derangement.

### Automated Analysis

**Figure 5:**
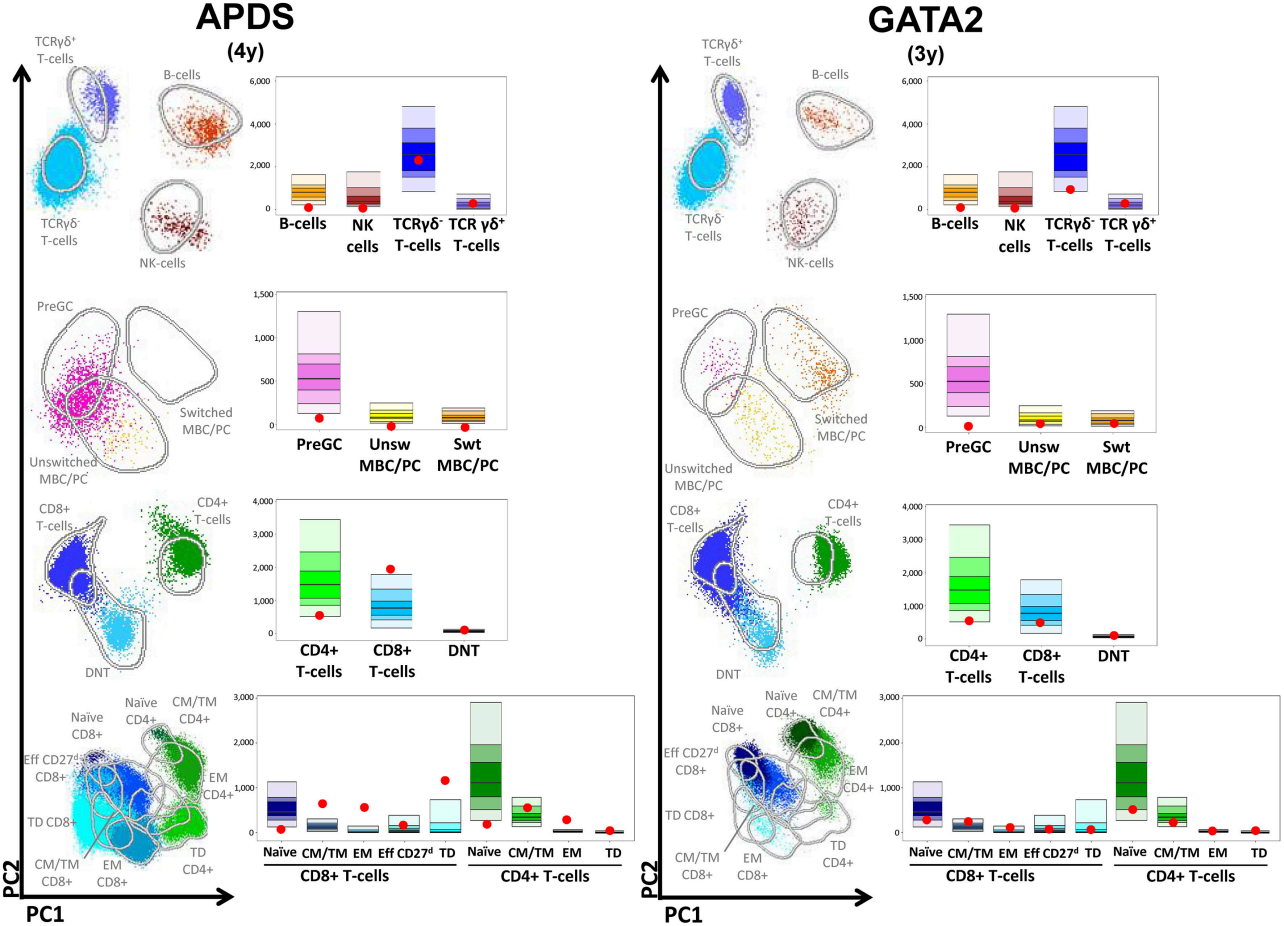
Multidimensional views of all lymphocyte subsets in combination with the absolute values of all subpopulations plotted as red dots in the age-matched reference bar graphs after automated analysis. **(APDS)** Patients with activated PI3K delta syndrome (APDS). (**GATA2**) Patient with GATA2 deficiency.

The advantage of the large reference data set of normal samples and well-annotated PID patient samples is that it can serve as templates for prospective data analysis. In the Infinicyt software program, an automatic analysis option has been included, which can be applied on all samples provided that the samples were processed, stained and measured according to the standardized EuroFlow protocols. Also in case the lyophilized version of the PIDOT is used, the data can be analyzed via this strategy (17). This new feature provides per patient the multidimensional APS plot, the absolute values of the lymphocyte subsets plotted in the age-matched bar graph and the numerical table with values for direct uploading in the electronic laboratory management and patient systems. For a patient with APDS and GATA2 the output is visualized in Figure 5. For the APDS patient this representation clearly shows the combination of low level of B-cells–preGC and especially (switched) memory B-cells, low naïve CD4 and CD8 cells and an expansion of the memory CD8 cell fractions, which is characteristic for APDS (18). Also for the GATA2-deficient patient displayed, the combined data show some characteristic features: reduced B and NK cells in combination with numbers of T-cell subsets which were below median but still within the normal range. Table 2 and Supplementary Tables 2, 3 can be consulted to verify how representative an immunophenotype is for a given genetically defined PID. This new application of EuroFlow software tools support diagnosis of PID.

## Discussion

In this study, we designed a single flow cytometry staining tube, PIDOT, for analysis of defined B and T cell subsets and validated its sensitivity performance on 250 healthy controls and 99 genetically-defined and IUIS-classified PID patients. This tube is fully standardized with the aim to have fully comparable data for international exchange of data and to support diagnosis of PID. With the multicenter EuroFlow approach, the PIDOT was designed over multiple rounds of design, testing in multiple labs, evaluation, and redesign. In addition, an application of new EuroFlow software tools with multidimensional pattern recognition was designed with inclusion of maturation pathways in multidimensional patterns (APS plots). Finally, we created a reference data base for automated data analysis, which can be implemented in diagnostic laboratories for routine diagnostics of patients suspected for PID. With this PIDOT, we could analyze all major lymphocyte subsets and the important lymphocyte subpopulations allowing the generation of data on the absolute counts and frequency of the lymphocyte subsets and a lymphocyte profile as APS view with a single tube. This tube can readily be implemented in the diagnostics of PID.

The PIDOT serves as the central tube in the EuroFlow algorithm for PID. Based on the results of the PIDOT other tubes shall be used for more detailed analysis of B- and/or T-cell subsets. For example, for patients with SCID the PIDOT is a strong screening tool, that in combination with the immunophenotyping of recent thymic emigrants in cases with present yet abnormal T-cells can provide diagnostic information with direct clinical consequences (Kalina et al. manuscript in preparation, in this issue). In the PIDOT tube we choose CD27 and CD45RA as a marker for naïve T cells (CD27+CD45RA+), while CD62L is used in the so called SCID-RTE tube together with CD45RO and CD31 to confirm the absence of recent thymic emigrants [Kalina et al. Manuscript in preparation, (19)]. Based on the flow cytometric results the treatment strategy can be initiated even prior to the result of genetic testing. The presence of T-cells in case of a clinical suspicion of SCID should be interpreted with care. It is known that especially RAG1 and RAG2 deficiencies can present with T-cells. The presence of T-cells in a patient clinically presenting as SCID can be due to hypomorphic mutation (with residual activity) (20) Alternatively, T-cells can be of maternal origin (21). In rare cases the presence of T-cells can be the result of reversion mutations (22). In these cases, we recommend additional labeling for SCID including CD31, CD62L, HLA-DR, and CD45RO for further typing of T-cells (Kalina et al. manuscript in preparation). For some PID disease categories, e.g., Common Variable Immunodeficiency (CVID) it might be necessary to have a more detailed phenotyping of the T- and B-cell subsets, e.g., with respect to certain subsets expressing specific Ig subclasses (8, 23).

If the PIDOT is used as screening tube to test whether the patient suffer from a PID, relative frequencies of naive CD4+ cells, as well as CD4 and CD8 effector memory cells were most frequently aberrant. We propose that this approach is used in any patient with a clinical suspicion of PID, because multiple and clear abnormal values are indicative of severe PID that requires adequate clinical management. In other, less pronounced phenotypes, PID screening tube can direct further evaluation including prioritization for NGS or gene panel evaluation). In PIDs that do not affect the lymphoid compartment (CGD, IRAK4 and complement deficiencies) no aberrant populations were identified, indicating that this tube is not useful for these categories. It will be of great value to prospectively collect the data of PID analyzed with the PIDOT to better define the characteristic pattern of aberrant subsets in a large cohort of genetically defined PID. Moreover, in combination with the clinical presentation and the exact mutation, the spectrum of PID can be better defined.

Recently, a lyophilized version of the PID screening tube has been developed and has proven to give the same results as when antibodies are used the liquid form and are added separately to the mixture. The advantage of using dried tubes is that it is not only time-saving and less prone to operational mistakes, but that it also significantly reduces the time spent in antibody inventory management (ordering of reagents and acceptance testing of antibodies (one single tube vs. 12 individual antibodies), including all the corresponding registrations (17), thus making the process suitable for any clinical laboratory. The major advantage of the EuroFlow approach using standardized protocols and flowcytometer instruments setting is that the generated data can be fully exchanged between laboratories and diagnostic centers, and will allow the generation of databases of patient that are extremely rare. Standardized EuroFlow multicolor flow cytometry is relatively easy to adopt, as EuroFlow has created standard operating protocols, published them on a eurofow.org website and commented on their use in the literature. In order to support the widespread adoption in reasonable quality EuroFlow educational meetings are organized as well as Quality Assessment (24).

Availability of the fully standardized PIDOT and accessibility to EuroFlow reference data base allows any lab in the world to perform standardized PID diagnostic, also in non-Western countries, because all over the world 8-color flow cytometers are now available, thanks to the HIV diagnostics and leukemia and lymphoma diagnostics. In addition, the multidimensional data analysis strategy and visualization will disclose new information, which is otherwise lost if only frequencies and absolute numbers of separate lymphocyte subsets are taken in consideration. With these developments, a new dimension is added to flow cytometry in the PID field in which the number of newly identified PIDs is still increasing.

## Author Contributions

MvdB, TK, MP-A, MvZ, AO, and JvD contributed to the conception and design of the study. TK, MP-A, MV, EL-G, EB, CB, AS, A-KK, MW, EM, VS, and JS performed the data acquisition and data analysis. MP-A and EB organized the database; MvdB, TK, AO, and JvD wrote the manuscript. All authors contributed to manuscript revision, read and approved the submitted version.

### Conflict of Interest Statement

JvD, MvdB, TK, MP-A, MV, EL-G, A-KK, MvZ, EB, and AO each report being one of the inventors on the EuroFlow-owned patent PCT/NL 2015/050762 (Diagnosis of primary immunodeficiencies), which is licensed to Cytognos, a company that pays royalties to the EuroFlow Consortium. JvD and AO report an Educational Services Agreement from BD Biosciences. The remaining authors declare that the research was conducted in the absence of any commercial or financial relationships that could be construed as a potential conflict of interest.
