# Supplementary material for: The EuroFlow PID Orientation Tube for Flow Cytometric Diagnostic Screening of Primary Immunodeficiencies of the Lymphoid System
Source: Front Immunol. 2019 Mar 4;10:246. doi: 10.3389/fimmu.2019.00246 (PMC6410673; doi:10.3389/fimmu.2019.00246)
Supplement: Supplementary file 1 [file Table_1.pdf]

Supplementary Table 1: Successive versions of the screening tube

Version	PacB/BV421	PacO/BV510	FITC	PE	PerCPCy 5.5	PECy7	APC	APC-A750
1	CD4+IgM PacB	CD45 PacO	CD8+smIgD	CD16 +CD56	CD27	CD19+TCRgd	CD3	CD38
2	CD27 BV421	CD45 PacO	CD8+smIgD	CD16 +CD56	CD4+IgM	CD19+TCRgd	CD3	CD38
3	CD27 BV421	CD45 PacO	CD8+smIgD	CD16 +CD56	CD4+IgM	CD19+TCRgd	CD3	CD45RA
4 (Final)	CD27 BV421	CD45RA BV510	CD8+smIgD	CD16 +CD56	CD4+IgM	CD19+TCRgd	CD3	CD45
