# Supplementary figures and images for: The EuroFlow PID Orientation Tube for Flow Cytometric Diagnostic Screening of Primary Immunodeficiencies of the Lymphoid System
Source: Front Immunol. 2019 Mar 4;10:246. doi: 10.3389/fimmu.2019.00246 (PMC6410673; doi:10.3389/fimmu.2019.00246)

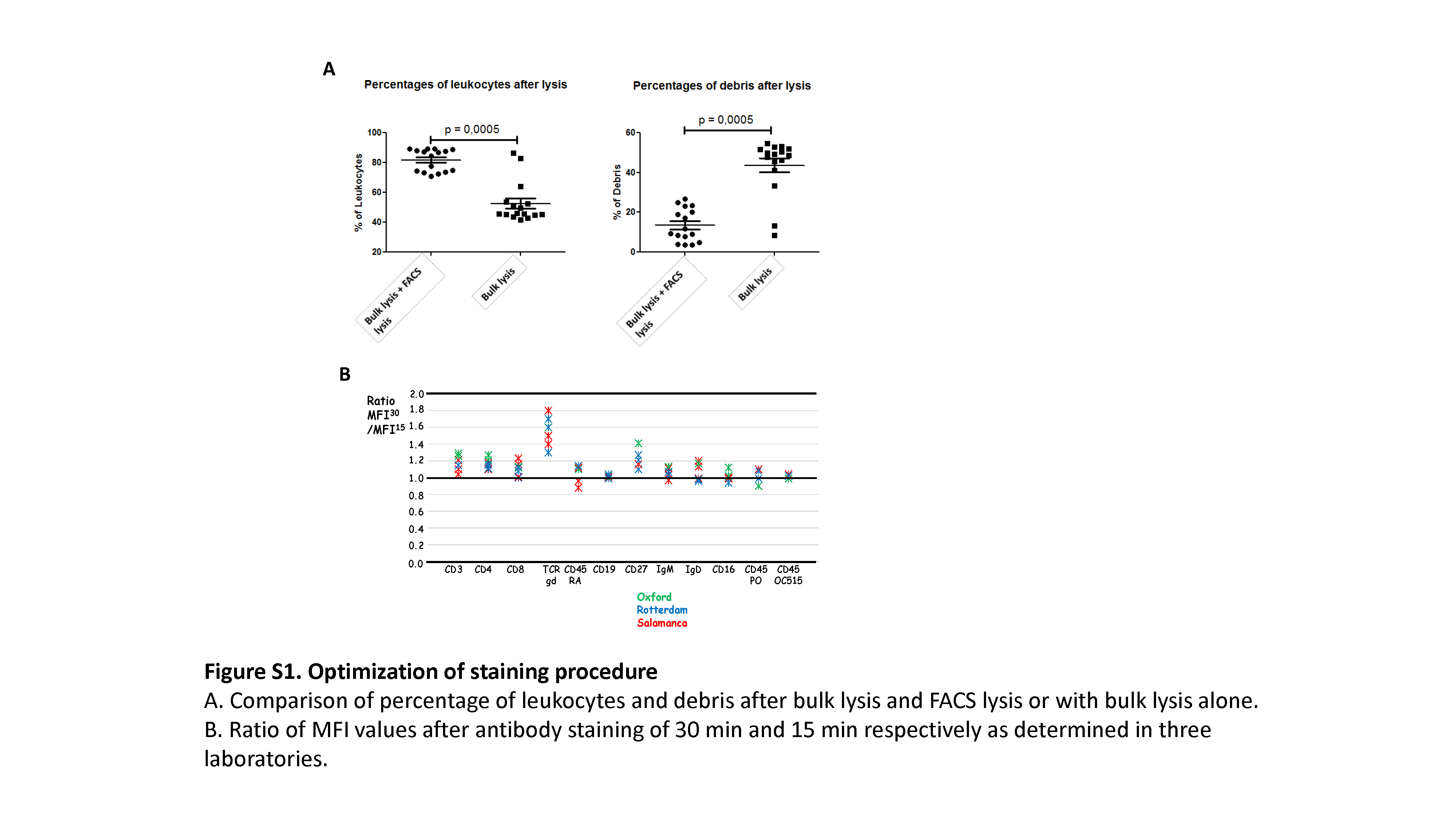

Supplement: Supplementary file 2 [file Image_1.TIFF]
