# Supplementary material for: The EuroFlow PID Orientation Tube for Flow Cytometric Diagnostic Screening of Primary Immunodeficiencies of the Lymphoid System
Source: Front Immunol. 2019 Mar 4;10:246. doi: 10.3389/fimmu.2019.00246 (PMC6410673; doi:10.3389/fimmu.2019.00246)
Supplement: Supplementary file 3 [file Image_2.pdf]

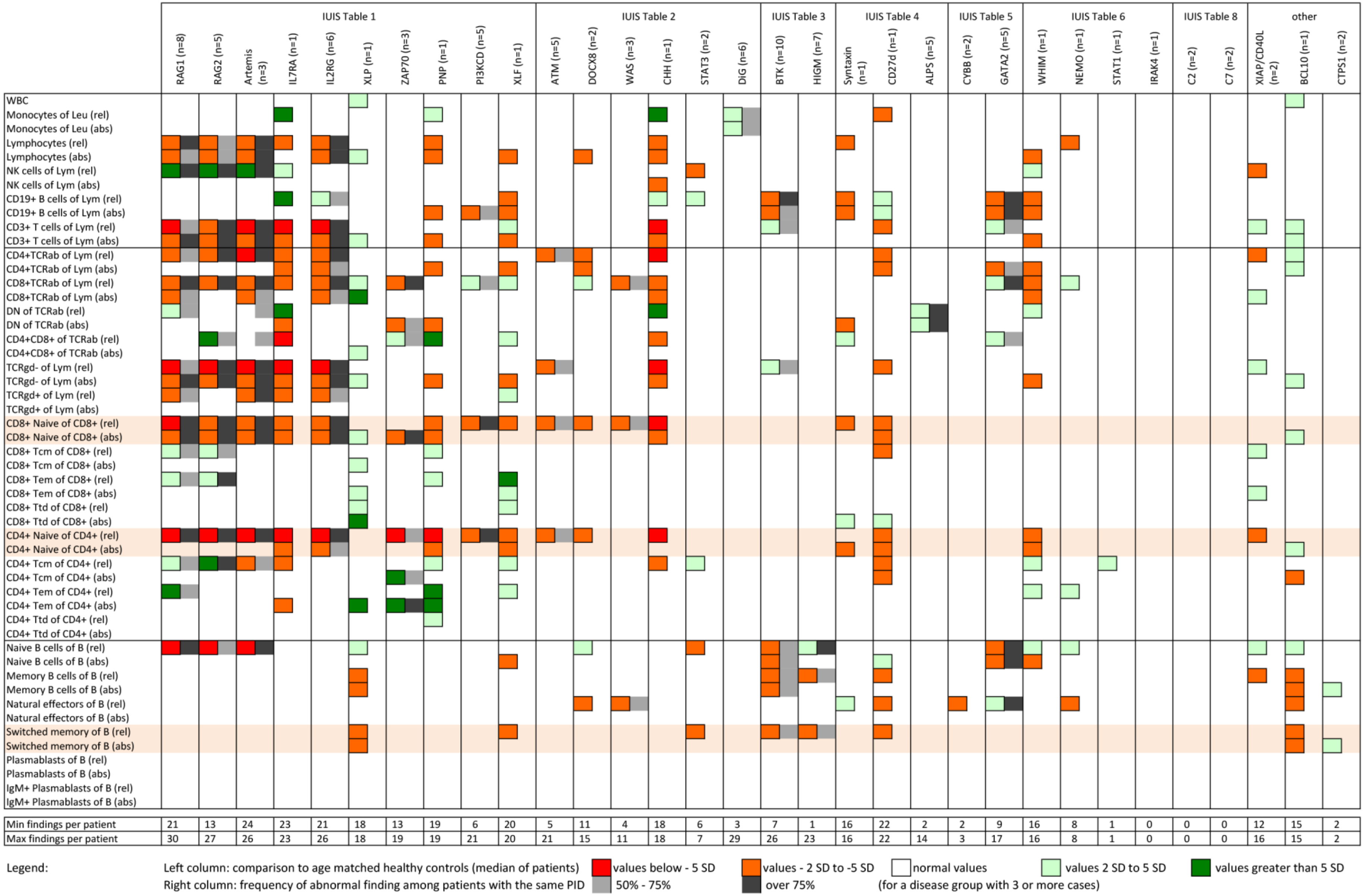

Figure S2. Discriminative power of the PIDOT. List of the 36 parameters determined with the EuroFlow screening tube. In the left columns the distance in number of standard deviation (SD) from controls of the same age group as the patient are given per parameter, followed by a scoring ranging from extremely high (>5SD) to high (>2SD), low (<-2SD) or extremely low (<-2SD). In the right column the frequency of abnormal findings among patients with the same PID are displayed for a category of > 3 cases per group in light grey (50-75%) and dark grey (>75%). The number of abnormal parameter per disease group was scored.
