# Supplementary material for: The EuroFlow PID Orientation Tube for Flow Cytometric Diagnostic Screening of Primary Immunodeficiencies of the Lymphoid System
Source: Front Immunol. 2019 Mar 4;10:246. doi: 10.3389/fimmu.2019.00246 (PMC6410673; doi:10.3389/fimmu.2019.00246)
Supplement: Supplementary file 4 [file Table_2.pdf]

Supplementary Table 2: Frequency of patients with inborn errors of immunity showing defects of the T-cells subsets identified in the EF PIDOT, as compared to age-reference values.

Disease	CD4^{+} TCRgd^{-} T-cells			CD8^{+} TCRgd^{-} T-cells			DNT TCRgd^{-} T-cells	TCRgd^{+} T-cells	Any of them
	Total	Naïve	CM	Total	Naïve	CM			
**SCID** (n=24)	88%	100%	67%	83%	100%	52%	0%	79%	100%
IL2Rg	6/6	6/6	4/6	5/6	6/6	2/6	0/6	5/6	6/6
IL7R	1/1	1/1	1/1	1/1	1/1	1/1	0/1	1/1	1/1
RAG1	6/8	8/8	4/6	7/8	8/8	3/6	0/8	7/8	8/8
RAG2	4/5	5/5	5/5	4/5	5/5	5/5	1/5	3/5	5/5
DCLRE1C	3/3	3/3	NA	3/3	3/3	NA	0/3	3/3	3/3
NHEJI	1/1	1/1	0/1	0/1	1/1	0/1	0/1	0/1	1/1
**CID** (n=12)	17%	17%	17%	25%	42%	17%	8%	0%	60%
CD40L	0/6	0/6	0/6	0/6	0/6	0/6	1/6	0/6	1/6
ZAP70	0/3	0/3	0/3	3/3	3/3	1/3	1/3	0/3	3/3
DOCK8	2/2	2/2	½	0/2	2/2	0/2	0/2	0/2	2/2
BCL10	0/1	0/1	1/1	0/1	0/1	1/1	0/1	0/1	1/1
**CID with syndromic features** (n=20)	25%	50%	5%	30%	55%	5%	15%	5%	70%
WASp	0/3	0/3	0/3	2/3	3/3	1/3	0/3	0/3	3/3
ATM	2/6	5/6	0/6	1/6	5/6	0/6	0/6	0/6	5/6
Di George	1/6	2/6	0/6	2/6	2/6	0/6	2/6	0/6	3/6
STAT3	0/2	1/2	0/2	0/2	0/2	0/2	1/2	0/2	1/2
NEMO	1/2	1/2	1/2	0/2	0/2	0/2	0/2	0/2	1/2
PNP	1/1	1/1	0/1	1/1	1/1	0/1	0/1	1/1	1/1
**PAD** (n=16)	13%	25%	13%	0%	25%	0%	0%	13%	31%
BTK	0/10	0/10	0/10	0/10	0/10	0/10	0/10	1/10	1/10
PI3KCD	2/5	4/5	2/5	0/5	4/5	0/5	0/5	1/5	4/5
AID	0/1	0/1	0/1	0/1	0/1	0/1	0/1	0/1	0/1
**Disease of immune dysregulation** (n=10)	0%	30%	10%	0%	20%	10%	40%	0%	70%
Syntaxin	0/1	1/1	0/1	0/1	1/1	0/1	0/1	0/1	1/1
FAS	0/5	1/5	0/5	0/5	0/5	0/5	4/5	0/5	5/5
XLP	0/1	0/1	0/1	0/1	0/1	0/1	0/1	0/1	0/1
CD27	0/1	1/1	1/1	0/1	1/1	1/1	0/1	0/1	1/1
CTPS1	0/2	0/2	0/2	0/2	0/2	0/2	0/2	0/2	0/2
**Defects of phagocytes or function** (n=10)	20%	10%	20%	0%	0%	10%	0%	0%	30%
CGD	1/5	0/5	0/5	0/5	0/5	0/5	0/5	0/5	1/5
GATA2	1/5	1/5	2/5	0/5	0/5	1/5	0/5	0/5	2/5
**Defects innate immunity** (n=3)	33%	67%	0%	33%	33%	33%	67%	33%	67%
STAT1	0/1	1/1	0/1	0/1	0/1	0/1	1/1	0/1	1/1
WHIM	1/1	1/1	0/1	1/1	1/1	1/1	1/1	1/1	1/1
IRAK4	0/1	0/1	0/1	0/1	0/1	0/1	0/1	0/1	0/1
**Complement deficiencies** (n=4)	0%	0%	0%	0%	0%	0%	0%	0%	0%

Results expressed as percentage of patients showing absolute counts below the lower limit of normality, compared to age-reference values obtained from 250 healthy donors analyzed with the same protocol, except for DNT TCRgd^{-} T-cells that is expressed as percentage of patients showing >6% from total TCRgd^{-} T-cells. SCID: Severe Combined Immunodeficiency. CID: Combined Immunodeficiency. PAD: Predominantly Antibody Deficiency. CM/TM: Central/Transitional Memory. NA: Not available.
