# Supplementary material for: The EuroFlow PID Orientation Tube for Flow Cytometric Diagnostic Screening of Primary Immunodeficiencies of the Lymphoid System
Source: Front Immunol. 2019 Mar 4;10:246. doi: 10.3389/fimmu.2019.00246 (PMC6410673; doi:10.3389/fimmu.2019.00246)
Supplement: Supplementary file 5 [file Table_3.pdf]

Supplementary Table 3: Frequency of patients with inborn errors of immunity showing defects of the B-cells subsets identified in the EF PIDOT, as compared to age-reference values.

Disease	PreGC	MBC/PC	Unswitched MBC/PC	Switched MBC/PC	Any of them
**SCID** (n=24)	**75%**	**100%**	**100%**	**100%**	**100%**
**IL2Rg**	**1/6**	**6/6**	**6/6**	**6/6**	**6/6**
**IL7R**	**0/1**	**1/1**	**1/1**	**1/1**	**1/1**
**RAG1**	**8/8**	**8/8**	**8/8**	**8/8**	**8/8**
**RAG2**	**5/5**	**5/5**	**5/5**	**5/5**	**5/5**
**DCLRE1C**	**3/3**	**3/3**	**3/3**	**3/3**	**3/3**
**NHEJI**	**1/1**	**1/1**	**1/1**	**1/1**	**1/1**
**CID** (n=12)	**8%**	**42%**	**42%**	**83%**	**83%**
**CD40L**	**0/6**	**1/6**	**1/6**	**6/6**	**6/6**
**ZAP70**	**0/3**	**1/3**	**1/3**	**1/3**	**1/3**
**DOCK8**	**1/2**	**2/2**	**2/2**	**2/2**	**2/2**
**BCL10**	**0/1**	**1/1**	**1/1**	**1/1**	**1/1**
**CID with syndromic features** (n=20)	**25%**	**45%**	**45%**	**45%**	**60%**
**WASp**	**0/3**	**3/3**	**3/3**	**1/3**	**3/3**
**ATM**	**3/6**	**4/6**	**3/6**	**4/6**	**4/6**
**Di George**	**1/6**	**1/6**	**1/6**	**1/6**	**1/6**
**STAT3**	**0/2**	**0/2**	**1/2**	**1/2**	**2/2**
**NEMO**	**0/2**	**1/2**	**1/2**	**1/2**	**1/2**
**PNP**	**1/1**	**0/1**	**0/1**	**1/1**	**1/1**
**PAD** (n=16)	**75%**	**94%**	**94%**	**94%**	**100%**
**BTK**	**10/10**	**10/10**	**10/10**	**10/10**	**10/10**
**PI3KCD**	**2/5**	**5/5**	**5/5**	**4/5**	**5/5**
**AID**	**0/1**	**0/1**	**0/1**	**1/1**	**1/1**
**Disease of immune dysregulation** (n=10)	**20%**	**40%**	**40%**	**50%**	**60%**
**Syntaxin**	**1/1**	**1/1**	**0/1**	**1/1**	**1/1**
**FAS**	**0/5**	**2/5**	**2/5**	**2/5**	**2/5**
**XLP**	**0/1**	**0/1**	**0/1**	**1/1**	**1/1**
**CD27**	**0/1**	**0/1**	**1/1**	**0/1**	**1/1**
**CTPS1**	**1/2**	**1/2**	**1/2**	**1/2**	**1/2**
**Defects of phagocytes or function** (n=10)	**20%**	**40%**	**30%**	**50%**	**60%**
**CGD**	**0/5**	**2/5**	**2/5**	**2/5**	**3/5**
**GATA2**	**2/5**	**2/5**	**1/5**	**3/5**	**3/5**
**Defects innate immunity** (n=3)	**33%**	**67%**	**67%**	**67%**	**67%**
**STAT1**	**0/1**	**1/1**	**1/1**	**1/1**	**1/1**
**WHIM**	**1/1**	**1/1**	**1/1**	**1/1**	**1/1**
**IRAK4**	**0/1**	**0/1**	**0/1**	**0/1**	**0/1**
**Complement deficiencies** (n=4)	**0%**	**0%**	**0%**	**0%**	**0%**

Results expressed as percentage of patients showing absolute counts below the lower limit of normality, compared to age-reference values obtained from 250 healthy donors analyzed with the same protocol. SCID: Severe Combined Immunodeficiency. CID: Combined Immunodeficiency. PAD: Predominantly Antibody Deficiency. MBC/PC: Memory B-cells/Plasma cells.
