# Supplementary material for: The EuroFlow PID Orientation Tube for Flow Cytometric Diagnostic Screening of Primary Immunodeficiencies of the Lymphoid System
Source: Front Immunol. 2019 Mar 4;10:246. doi: 10.3389/fimmu.2019.00246 (PMC6410673; doi:10.3389/fimmu.2019.00246)
Supplement: Supplementary file 6 [file Table_4.pdf]

Supplementary Table 4: Reference values for absolute counts of peripheral blood leukocytes subpopulations calculated with the Euroflow PID orientation tube in healthy controls per age group.

	CB (n=15)	NB (n=16)	1-5 m (n=12)	6-11 m (n=7)	12-24 m (n=30)	2-4 y (n=35)	5-9 y (n=28)	10-17 y (n=18)	18-29 y (n=31)	30-39 y (n=15)	40-49 y (n=12)	50-59 y (n=10)	60-69 y (n=10)	>70y (n=11)
**Lymphocytes**	2040-5688	1818-8667	2443-9955	4821-8531	2356-13275	1620-6856	1827-4564	1238-4792	895-3684	877-3119	1139-3192	1836-2977	1531-4891	1795-3934
**Total B-cells**	347-1053	108-961	470-4327	896-2316	353-2300	232-1637	157-725	173-1194	56-470	41-391	48-314	90-413	68-384	42-242
**PreGC B-cells**	347-1053	108-955	446-3911	652-1956	300-2255	175-1348	70-573	107-1040	22-291	14-298	29-195	33-265	29-302	16-233
**PostGC B-cells**	0-0	0-6	6-415	94-361	45-466	44-491	68-276	35-173	18-232	23-176	19-192	39-160	39-145	9-81
**Unswt PostGC**	0-0	0-2	3-356	32-272	23-280	22-333	23-155	18-89	7-124	8-76	10-129	14-84	22-79	2-33
**Swt PostGC**	0-0	0-4	3-59	18-103	23-187	21-199	36-182	14-101	11-125	12-121	9-63	25-101	13-65	7-56
**T-cells**	1186-4113	1476-8327	1680-7754	3764-6289	1900-9345	852-5333	1352-3275	930-3477	564-2935	612-2156	743-2316	1413-2379	1110-2941	636-3030
**CD4+CD8- T-cells**	952-3097	1092-5341	1273-5633	2093-4769	617-5959	516-3448	776-1815	576-1891	207-1900	384-1303	501-1654	815-1769	345-1386	410-1474
**Naïve CD4+CD8-**	920-2897	1092-5341	1092-5337	1748-4201	360-5273	276-2902	424-1393	264-1484	74-1173	89-683	83-676	196-1057	75-643	27-939
**CM/TM CD4+CD8-**	12-199	0-692	0-684	233-630	252-1135	152-802	206-565	244-593	117-886	229-616	235-589	360-784	238-764	173-738
**EM CD4+CD8-**	0-0	0-3	2-37	5-33	3-71	9-66	10-95	27-222	14-500	14-211	25-137	25-208	30-476	36-539
**TD CD4+CD8-**	0-0	0-0	0-8	0-4	0-6	0-43	0-79	0-46	0-87	0-38	0-663	0-356	0-92	0-219
**CD4-CD8+ T-cells**	213-1138	340-2827	354-2006	720-1271	364-2498	188-1805	366-1171	261-1189	160-1103	198-889	133-719	194-1432	316-1520	202-1571
**Naïve CD4-CD8+**	200-1010	340-2827	330-1841	564-1040	222-2178	126-1130	175-730	94-986	33-737	37-325	29-386	65-223	5-223	2-165
**CM/TM CD4-CD8+**	7-166	0-226	24-500	59-215	34-882	25-309	62-456	76-427	54-422	87-424	59-348	78-453	97-321	42-305
**EM CD4-CD8+**	0-0	0-0	0-146	1-37	0-129	0-151	1-51	6-62	5-69	2-515	6-115	2-323	11-363	1-925
**CD27+ TD CD4-CD8+**	0-0	0-0	0-432	0-100	0-238	0-385	0-83	3-93	0-144	1-95	7-118	16-457	4-173	17-576
**CD27- TD CD4-CD8+**	0-0	0-0	0-255	0-66	0-826	0-735	0-325	3-284	1-240	1-273	0-196	8-500	0-905	12-736
**CD4-CD8- TCRgd- T-cells**	0-37	2-171	9-66	15-73	20-141	3-104	13-80	8-53	5-79	6-26	4-24	6-21	4-27	2-14
**CD4-CD8- TCRgd+ T-cells**	18-121	25-162	25-435	128-335	86-537	44-784	66-416	56-332	11-470	11-173	33-231	7-99	22-251	4-1060
**NK cells**	200-1305	73-721	167-1359	237-1146	104-2436	138-1759	106-1348	109-1021	81-615	130-739	161-672	150-433	195-1737	124-918
